# Aortic Pseudoaneurysm Formation Following Concurrent Chemoradiotherapy And Metallic Stent Insertion in a Patient With Esophageal Cancer

**DOI:** 10.1097/MD.0000000000000862

**Published:** 2015-05-22

**Authors:** Pei-Yu Hou, Chung-Jen Teng, Chen-Shuan Chung, Chao-Yu Liu, Chun-Chieh Huang, Miu-Hsiang Chang, Pei-Wei Shueng, Chen-Hsi Hsieh

**Affiliations:** From the Division of Radiation Oncology, Department of Radiology, Far Eastern Memorial Hospital, New Taipei City (P-YH, P-WS, C-HH); Department of Medicine (C-JT, C-HH); Institute of Traditional Medicine, School of Medicine, National Yang-Ming University, Taipei (C-HH); Division of Oncology and Hematology, Department of Medicine, Far Eastern Memorial Hospital, New Taipei City (C-JT); Institute of Public Health, National Yang-Ming University, Taipei (C-JT); Department of Internal Medicine, Far Eastern Memorial Hospital, New Taipei City (C-SC); College of Medicine, Fu Jen Catholic University, New Taipei City (C-SC); Department of Thoracic surgery, Far Eastern Memorial Hospital, New Taipei City (C-YL); Department of Medical Imaging, Far Eastern Memorial Hospital, New Taipei City (C-CH); Department of Anatomical Pathology, Far Eastern Memorial Hospital, New Taipei City (M-HC); Department of Radiology, Tri-Service General Hospital, National Defense Medical Center, Taipei, Taiwan (P-WS).

## Abstract

Aortic pseudoaneurysm formation subsequent to concurrent chemoradiotherapy (CCRT) for esophageal cancer patient with esophageal metallic stent insertion is a rare condition.

A 52-year-old man with esophageal cancer, cT4N1M0, stage IIIC, was treated with concurrent weekly cisplatin (30 mg/m^2^) and 5-Fluorouracil (500 mg/m^2^) as well as radiotherapy (50.4 Gy in 28 fractions) for 6 weeks. An esophageal metallic stent was inserted for dysphagia 1 week after initiation of CCRT. During the treatment regimen, the platelet count dropped to less than 200 × 10^3^ /μL. One month after the completion of CCRT, chest CT revealed the presence of an aortic pseudoaneurysm as well as aortoesophageal fistulas. A thoracic aortic endografting was performed and the patient responded well to surgery. However, the patient died 2 months later due to a nosocomial infection.

Multimodality treatment for esophageal cancer comprising cisplatin-based CCRT and esophageal metallic stent placement near a great vessel may increase the risk of pseudoaneurysm formation.

## INTRODUCTION

Treatment options for patients with esophageal cancer and malignant dysphagia include endoluminal stenting, surgery, radiotherapy, brachytherapy, chemotherapy, and chemoradiotherapy (concurrent chemoradiotherapy, CCRT).^1,2^ Eldeeb and El-Hadaad^3^ showed that radiotherapy combined with stenting offers a survival advantage. However, metallic stents have been shown to cause irradiation dose perturbation between the stent and surrounding tissues, resulting in intimal thickening, proteoglycan deposition and increased inflammatory cell content, thereby increasing the risk of vascular disease development in cancer patients who undergo RT.^4,5^

Cisplatin (CDDP) and 5-Fluorouracil (5-FU) are reported to be the most active chemotherapeutic regimens for patients with esophageal cancer.^6^ Both drugs are radiosensitizors that enhance the cytotoxic effect of irradiation.^7,8^ Additionally, they also cause the damage of the arterial endothelium.^9,10^

Aortic arch pseudoaneurysm is a rare condition but carries a high risk of rupture. Neoadjuvant chemoradiotherapy followed by esophagectomy caused aortic fistula and related to sudden death for esophageal carcinoma had been reported.^11^ However, pseudoaneurysm development as a result of intraluminal stenting and CCRT in patients with esophageal cancer has never been reported. Herein, we present a case of aortic pseudoaneurysm development in a patient who underwent CCRT for esophageal cancer and metallic stent placement for cancer-induced dysphagia.

## CASE REPORT

**FIGURE 1:**
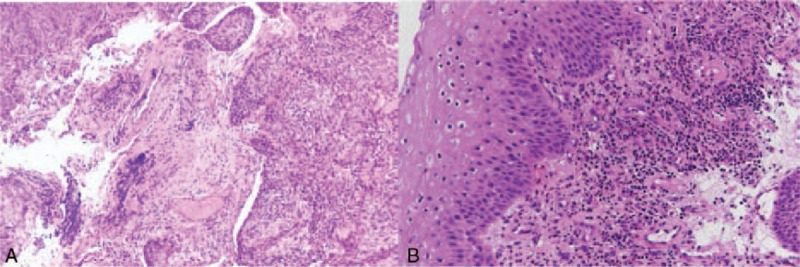
(A) Histopathologic analysis revealed predominantly small blue round cells with invasive nests that had grown beyond the mucosa (hematoxylin and eosin stain, magnification ×100), findings suggestive of squamous cell carcinoma. (B) Representative hematoxylin eosin staining after chemoradiation therapy (magnification ×100). Note the large number of leukocytes, the marked accumulation of eosinophilic nucleoli, mixed inflammation, and debris in the mucosal layer.

**FIGURE 2:**
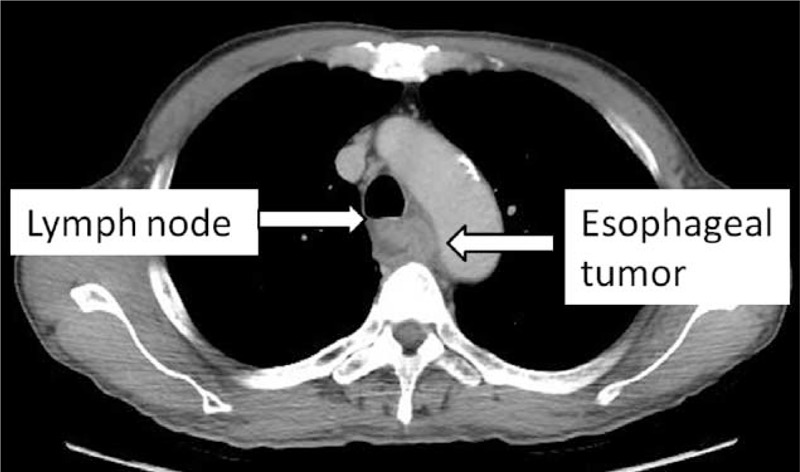
Chest computed tomography (CT) shows an esophageal tumor with obstruction at the middle third portion of the esophagus invading the adventitia. Also note the enlarged lymph node between the esophagus and the distal trachea.

**FIGURE 3:**
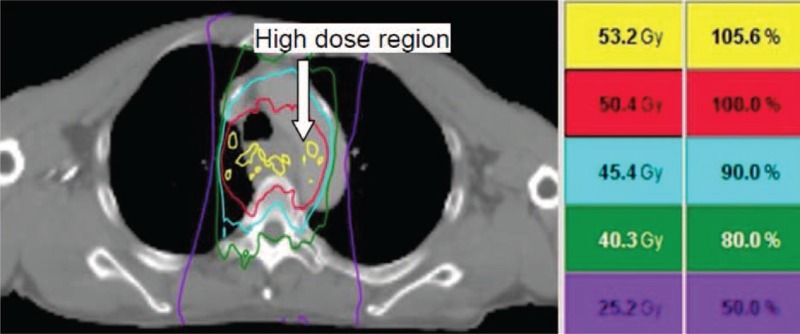
The dose distribution in the tumor and surrounding tissues. The high-dose regions with radiation dose of 53.2 Gy are adjacent to the wall of the aorta.

**FIGURE 4:**
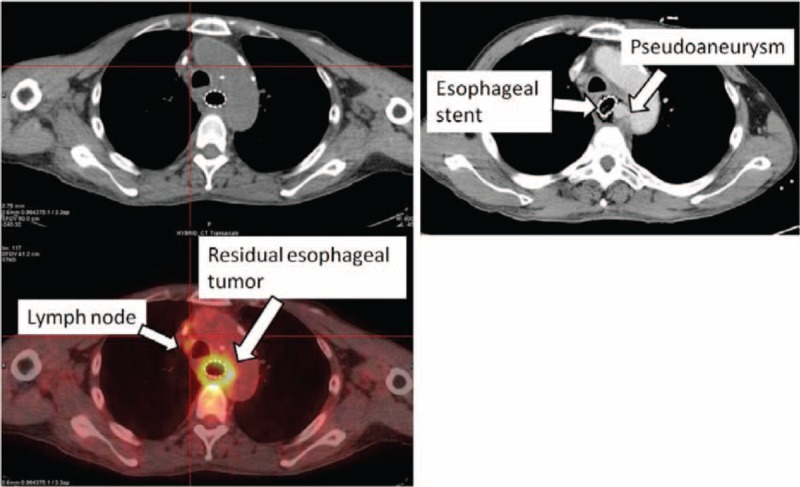
(A) Follow-up positron emission tomography scan 1 month after the completion of concurrent chemoradiotherapy revealed a residual esophageal tumor and enlarged lymph nodes with intact aortic vessel. (B) Chest CT shows a pseudoaneurysm arising from the medial aspect of the proximal descending aorta.

A 52-year-old man, with history of hypertension, was diagnosed with stage IIIC (cT4N1M0, American Joint Committee on Cancer, 7th Edition) squamous cell carcinoma at the middle third of the esophagus (Figures 1A and 2) underwent neoadjuvant CCRT comprising concurrent weekly CDDP (30 mg/m^2^) and 5-FU (500 mg/m^2^) as well as radiotherapy (50.4 Gy in 28 fractions) for 6 weeks (Figure 3). The regimen in the clinical trial of Cancer and Leukemia Group B (CALGB 9781) was 4-weekly schedule of chemotherapy.^6^ However, the regimen would be modified to be weekly regimen^12^ for patients who could not be treated in hospitalization or with poor compliance. Therefore, 4-weekly and weekly schedule both were the standard CCRT protocols in our hospital for esophageal cancer patients. Retrospective data were collected with the approval of the Institutional Review Board of Far Eastern Memorial Hospital (FEMH-IRB-103174-C). Approximately 1 week after beginning the CCRT regimen, however, the patient reported having difficulty swallowing semi-liquid food. A WallFlex™ esophageal stent (18 mm × 123 mm, Boston Scientific Corporation, MA) with metallic radiopaque material that is formed into a cylindrical mesh and was partially covered with a translucent silicone polymer to restrict tumor ingrowth through the wire mesh. After the stent was inserted, the CCRT regimen was continued. Platelet count at the time of stent insertion was 234 × 10^3^/μL. In the following weeks, the platelet counts ranged from 91 × 10^3^ to 153 × 10^3^/μL. One month after completion of the CCRT regimen, histopathologic analysis of biopsy specimens of the esophageal tumor revealed inflammation with ulceration change (Figure 1B) and positron emission tomography (PET)–computed tomography (CT) showed a residual esophageal tumor and persistently enlarged lymph nodes with intact aortic vessel (Figure 4A). However, a few days after the PET-CT study the patient developed midthoracic pain and a hemorrhagic cough. Chest CT at the emergency department revealed a pseudoaneurysm arising from the medial aspect of the proximal descending aorta (Figure 4B). Massive hematemesis was subsequently noted. Emergency surgery was performed to repair the pseudoaneurysm. During the surgery, aorto-esophageal fistulas with active bleeding located about 2 cm distal to the left subclavian artery orifice were noted. Aortography revealed no evidence of rupture at the descending aorta. A thoracic aortic endografting with an aortic stent (8.7 cm in length) was performed and the patient responded well to surgery. One month later, he received adjuvant chemotherapy with CDDP (70 mg/m^2^) and 5-FU (1000 mg/m^2^) for residual esophageal cancer (Figure 4A). However, the patient developed symptoms of fever and cough with leukocytosis. The chest X-ray revealed increased infiltrations over left lower lung. Hospital-acquired pneumonia was impressed, and empirical antibiotics were prescribed firstly. The culture of sputum and blood were negative findings, respectively. Unfortunately, progressive pneumonia with septic shock and respiratory failure was noted even through aggressive treatment. Patient expired 2 months after the thoracic aortic endografting. Autopsy was suggested. However, patient's families want to maintain the patient's body completely without surgery procedure especially after patient expired. To obey the wish of family, autopsy was not conducted for determining the reason of the patient's death.

## DISCUSSION

A pseudoaneurysm is an outpouching of a vessel involving a defect of the tunica intima and media that contains a hematoma.^13^ Numerous causes of pseudoaneurysm formation have been reported, including infection, traumatic reaction, iatrogenic injury, suture dehiscence or loosening, infiltrating neoplasms, tissue necrosis, and a low platelet count (<200 × 10^3^ /μL).^14–16^

Radiotherapy is a well-known cause of vascular disease especially when vessels are exposed to radiation doses ranging from 25 to 40 Gy.^17^ In our patient, the aortic pseudoaneurysm developed in a region that received a total radiation dose of 53.2 Gy (Figures 3 and 4B). The metallic stent irradiated that could result in an overdose of 14% to 21% at a depth of 0.5 mm in the esophageal wall.^18^ Nevertheless, the dose perturbation caused by the backscatter of WallFlex™ esophageal stent ranges from 6% to 13%,^4^ resulting in an effective dose of 56.5–60.3 Gy for the patient reported here. These influences might contribute to better tumor eradication but increases the probability of vascular changes.

Radiotherapy also increases the production of free radicals and oxidative stress, processes that upregulate numerous pathways pertinent to vascular disease, including matrix metalloproteinases (MMP), adhesion molecules, pro-inflammatory cytokines, and smooth muscle cell proliferation and apoptosis, while inactivating vasculoprotective nitric oxide (NO).^19^ Furthermore, fractionated doses increase the adhesiveness of aortic endothelial cells through chemokine-dependent signaling from endothelial cells to leukocytes and significantly increase intercellular adhesion molecule 1 (ICAM-1) mRNA and endothelial adhesiveness, which also accelerate vascular inflammation.^20,21^

Interestingly, neutrophils localized at inflammatory sites can potentially degrade collagen by releasing members of the MMP family.^22^ Moreover, histological examination has shown that radiotherapy can induce intimal thickening and inflammatory cell infiltrates.^5^ Histologic examination of biopsy specimens from our patient also disclosed neutrophil accumulation and mixed inflammation in the mucosal layer after CCRT (Figure 1B). Papalambros et al^23^ found that the concentration of MMP-9 in the arterial wall correlates positively with aneurysm size and radiotherapy has been shown to increase the expression of MMP-9.^24^ Wilson et al^25^ found that the concentration of MMP-8 is increased at the site of abdominal aortic aneurysm rupture, and radiotherapy also enhances the expression of MMP-8, which modulates the pharmacokinetics of anticancer drugs.^26^

CDDP and 5-FU are radiosensitizors that enhance the cytotoxic effects of radiotherapy.^7,8^ Although CCRT is more effective at tumor eradication than radiotherapy alone, the combined use of CDDP, 5-FU, and radiotherapy can cause unpredictable localized and systemic vascular inflammation with a higher incidence of atherosclerotic disease, coronary artery disease, and myocardial infarction.^9,10^ A previous study showed that CDDP results in a decrease in NO production and the upregulation of ICAM-1 expression in endothelial cells that accelerate vascular inflammation.^20,21,27^ Furthermore, an experimental study in rabbits showed that damage to the intima caused by 5-FU occasionally results in thrombus formation.^10^ Cwikiel et al^28^ found that the severity of endothelial injury was proportional to the incidence of thrombus formation. It could be reasonably hypothesized that CDDP-based regimens concurrent with radiotherapy not only sensitize the treatment effects of radiotherapy but also synchronize endothelial injury.

Aortic degeneration and atherosclerosis are the major causes of aotic aneurysms.^29^ Hypertension is a well-known predisposing factor for vascular degeneration and atherosclerosis. Vardulaki et al^30^ found that hypertension was an important risk factor for the development of abdominal aortic aneurysm, which increased the risk by 30% to 40%. Also, the prevalence of hypertension was over 60% in populations with thoracic aortic aneurysm.^31^ The degradation of extracellular matrix proteins by proteases like MMP has the primary role for vascular degeneration and atherosclerosis, and will weaken the aortic wall.^29^ Combined with the increased concentration and enhanced expression of MMP by RT, and vascular inflammation correlated to the radiosensitizors of CDDP and 5-FU, the underlying hypertension of our patient may accentuate the impairment of aortic wall, and probably increase the incidence of aneurysms formation.

The insertional procedure of metallic stent might contribute to the traumatic injury of aortic and esophageal walls to form the fistula. Schweigert et al^32^ found that among 17 patients with anastomotic leakage after esophagectomy who underwent endoscopic esophageal stent implantation, 18% developed aortic erosion followed by aortoesophageal fistula. Homann et al^33^ revealed that patients with esophageal metallic stent implantation experienced esophageal wall and respiratory tract impairment, and the incidence of delayed complications of esophagorespiratory fistula was 9%.

A platelet count below 200 × 10^3^/μL has been shown to be a predictor of iatrogenic pseudoaneurysm after percutaneous endovascular procedures.^15^ In our patient, platelet counts ranged from 91 to 153 × 10^3^/μL 2 weeks after initiation of CCRT that perhaps increased the incidences of iatrogenic psuedoaneurysm also.

## CONCLUSION

Multimodality treatment for esophageal cancer comprising CDDP-based CCRT and esophageal metallic stent placement near a great vessel may increase the risk of pseudoaneurysm formation.
